# Prevalence of pathological arrhythmia in patients triaged to “cardiac arrhythmia” in the emergency department: a preliminary study

**DOI:** 10.1186/s12245-022-00453-1

**Published:** 2022-09-13

**Authors:** Julia Novotny, Matthias Michael Klein, Magda Haum, Stephanie Raphaela Fichtner, Manuela Bernadette Thienel

**Affiliations:** 1 Department of Medicine I, University Hospital, LMU Munich, Marchioninistrasse 15, 81377 Munich, Germany; 2 Department of Neurology, University Hospital, LMU Munich, Marchioninistrasse 15, 81377 Munich, Germany; 3 Emergency Department, University Hospital, LMU Munich, Marchioninistrasse 15, 81377 Munich, Germany

**Keywords:** Cardiac arrhythmia, Emergency department, Atrial fibrillation, Emergency management

## Abstract

**Background:**

Symptoms caused by cardiac arrhythmia are common problems that lead to presentation to the emergency department. However, the prevalence of pathological heart rhythm in patients triaged for cardiac arrhythmia in the emergency department remains up to now unknown.

**Methods and results:**

In this retrospective study, patients triaged for cardiac arrhythmia admitted to the interdisciplinary emergency department of the Ludwig-Maximilians University Hospital in Munich within 1 year were included. Subsequently, cardiac rhythm in the 12-lead electrocardiogram, clinical presentation, admission rate, and diagnosis at discharge was analyzed. A total of 558 out of 39,798 patients were triaged for cardiac arrhythmia. Of these 42.3% of patients showed a pathological heart rhythm on the initial electrocardiogram (66.9% atrial fibrillation, 16.5% atrial flutter, 16.5% others). About 80% presented in emergency severity index III (many resources are needed without critical vitals) conditions. Sixty-two percent of the pathological electrocardiogram group and 60% of the sinus rhythm group of patients were admitted to the hospital, and 34.7% with pathological electrocardiogram underwent invasive investigations (16.8% in the sinus rhythm group). In 43.4% of patients, the diagnosis of cardiac arrhythmia was already known from previous medical contacts.

**Conclusion:**

A total of 1.8% of patients who presented to our interdisciplinary emergency department were triaged for cardiac arrhythmia. With 49.5%, the hospital admission rate was quite high but the patients presented to the emergency department in our cohort were rarely in critical condition. As a high percentage of our cohort had a history of cardiac arrhythmia, better outpatient management is needed for these patients to reduce emergency department visits and save resources.

## Background

Triage is a crucial process in the management of modern emergency departments (ED). Due to the limited amount of resources and the fluctuating quantity of admissions, not all patients can be treated immediately or simultaneously [1]. Therefore, patients with life-threatening illnesses or injuries must be reliably identified within a short time after arrival. Structured triage systems, e.g., the emergency severity index (ESI) are in use in many hospitals to evaluate the urgency of medical treatment [2]. To further improve processes in ED, patients might be allocated to certain triage codes according to the symptoms they describe to the emergency nurse at admission. One of these triage codes is “cardiac arrhythmia” (CA), a common cause of emergency consultation, particularly among the elderly [3]. In 2018, the prevalence of any abnormal heart rhythm in the general population in Great Britain was rated 2.35% and increased with age culminating in almost 5% of affected individuals aged over 65 years [4]. Along this line, the rate of hospitalization due to cardiac arrhythmia was over 500 of 100,000 inhabitants in Germany [5]. However, there is little information about the patient population pooled under the triage code CA in the ED setting. A detailed characterization of these patients is necessary to assess the urgent patients who need medical treatment and the expected effort within the ED. Besides improving processes and quality of medical management in the ED, this knowledge is also crucial regarding admission rates and occupancy of the hospitals. Therefore, this study aimed to analyze the number of patients and the actual prevalence of pathological heart rhythm in these patients and to evaluate further medical therapy, subsequent hospitalization, and main diagnosis at discharge.

## Methods

**Fig. 1:**
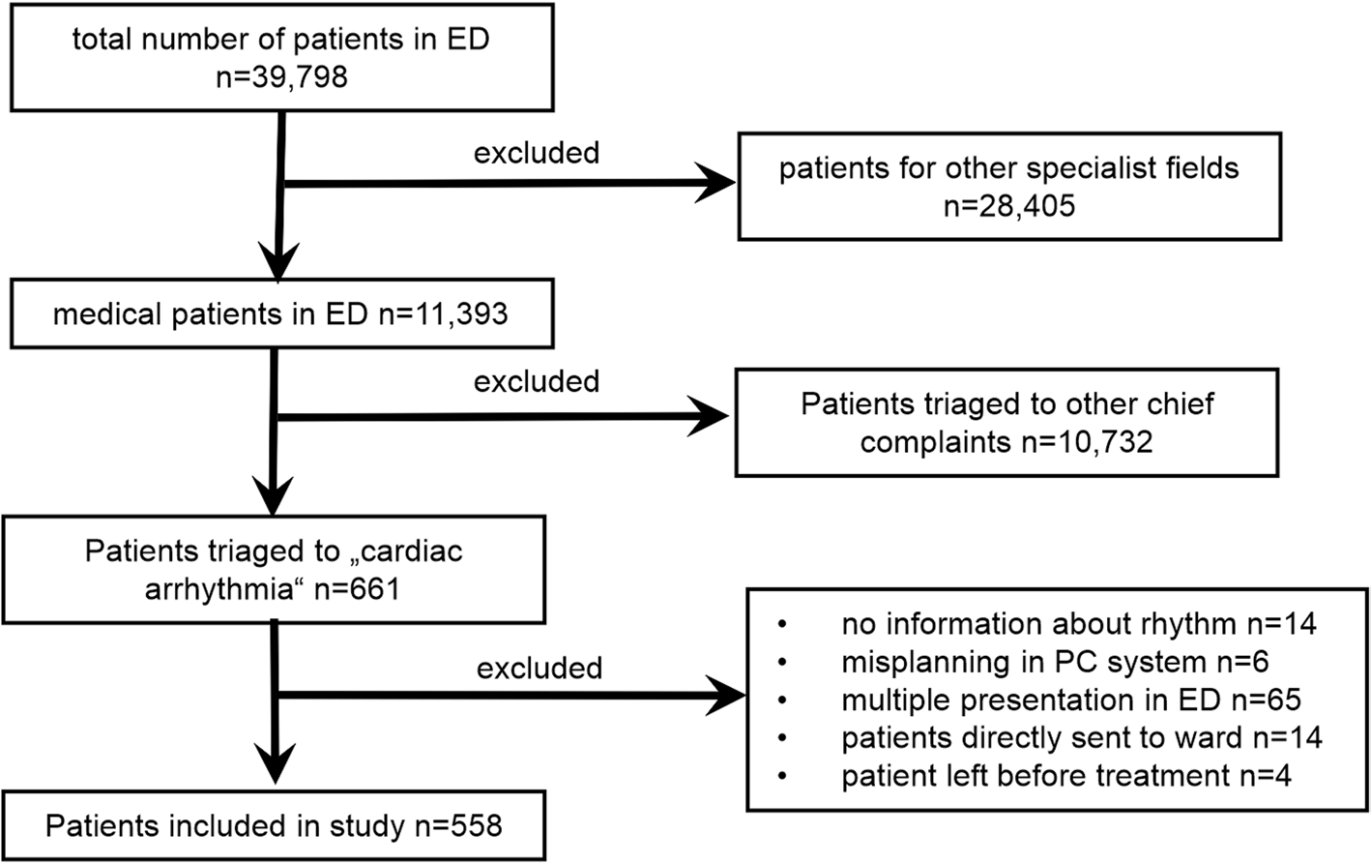
Study flow chart of patients’ inclusion in our study

Between November 2016 and October 2017, all patients triaged to CA in the interdisciplinary ED of the Ludwig-Maximilians University Hospital in Munich (Germany) were included in this retrospective study. We excluded patients, where no information about the first ECG was available, erroneously in the admission system entered patients, patients directly sent to the ward without treatment in the ED, and patients who left the hospital on their responsibility before treatment. When patients repetitively presented at the ED only the first visit was analyzed. (Fig. 1). The study conformed to the 1975 Declaration of Helsinki and was approved by the Ethics Committee of the Ludwig-Maximilians-University Munich. Data were retrieved from the two hospital information systems (EPIAS (epias GmbH, Idstein, Germany) and i.s.h.med for SAP (Cerner Deutschland)) and pseudonymized for analysis. Immediately after presentation at the ED, patients were routinely categorized according to the kind and severity of their symptoms by experienced nurses. Patients complaining about palpitations, tachycardia, or bradycardia were triaged to the code “cardiac arrhythmia.” Furthermore, ESI code [2] was used to evaluate the urgency of medical treatment (ESI (1) patient requires immediate life-saving intervention; ESI (2) high-risk situation or patient confused, lethargic, disorientated, or in severe pain/distress; ESI (3) many resources are needed without critical vitals; ESI (4) one resource is needed; ESI (5) no resource is needed). After this primary triage, patients were then treated according to the attending physician (e.g., anamnesis, physical examination, blood analysis, electrocardiogram (ECG)). Echocardiography workup was performed at the discretion of the treating physician on a total of 337 patients either in the ED or after admission. Examinations were performed by cardiologic residents or fellows trained in echocardiography. For further analysis, patients were then grouped into patients with normofrequent sinus rhythm (SR) or “pathological heart rhythm” using the first electrocardiogram (ECG) after presentation. Normofrequent SR was defined as SR with a heart rate of 50–100 beats per minute as patients within this range normally do not show any clinical signs of bradycardia or tachycardia requiring medical treatment. Pathological heart rhythm was defined as every heart rhythm different from normofrequent SR. Patients with signs of acute ischemia, i.e., ST-segment elevation and new left bundle branch block were immediately transferred to the catheter lab and excluded from this study. Baseline characteristics, mode of presentation, therapy, admission rate, and discharge diagnosis according to the ICD-10 classification were evaluated. Here, we only show discharge diagnoses of hospitalized patients since ICD codes of outpatients are monetarily irrelevant in Germany, and they were not audited by health insurance and were thus considered not reliable enough for analysis in our study. Altogether, 31 patients with indications for in-patient treatment were transferred to surrounding hospitals. In 5 patients, information about the admitting hospital is lacking. Sixteen of them were admitted to first-level hospitals because they did not need highly specialized technical equipment or intensive care. The residual 10 patients were transferred to second-level hospitals mostly because of missing bed capacity. Therefore, data regarding in-patient care or diagnosis at discharge refers only to patients admitted to our clinic.

The statistical analysis was conducted using GraphPad Prism® software (GraphPad Software Inc). D’Agostino-Pearson omnibus test was used to test the normality of data distribution. Normally distributed data were tested using the unpaired *t* test and non-normally distributed data using the Mann-Whitney *U* test. For comparison of categorical data, Fisher’s exact test was used. Data are mean ± standard deviation (SD). *P* values below 0.05 were considered significant.

## Results

Between November 2016 and October 2017, a total of 39,798 patients attended the interdisciplinary ED of the Ludwig-Maximilians University Hospital in Munich. 661 patients described one of the symptoms palpitations, bradycardia, or tachycardia and were therefore triaged to CA by the emergency nurse. For several reasons shown in Fig. 1, 103 patients had to be excluded, leaving 558 patients for analysis.

**Fig. 2:**
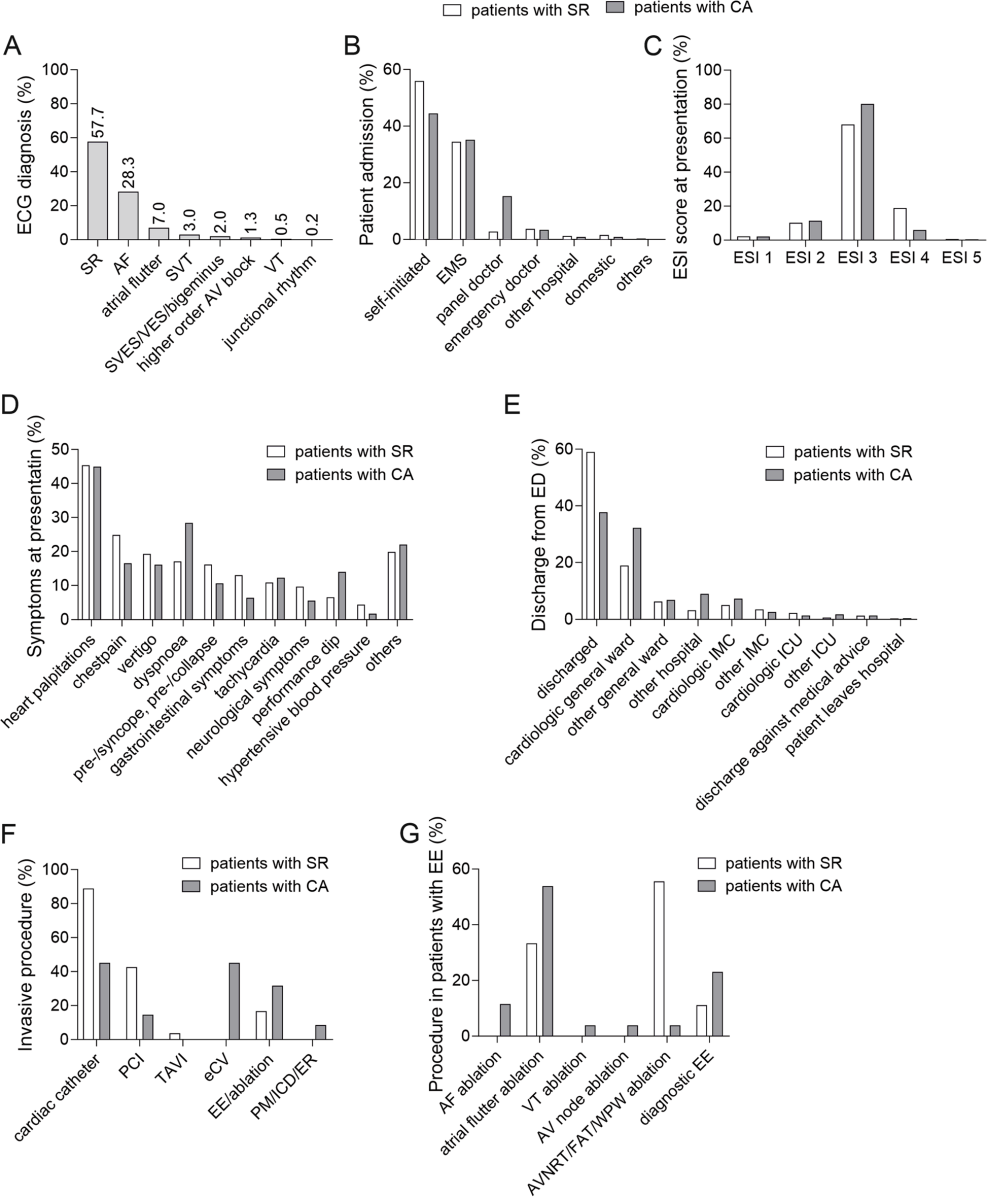
Clinical presentation. **A** Electrocardiogram diagnosis of patients triaged to cardiac arrhythmia (SR, sinus rhythm; AF, atrial fibrillation; SVT, supraventricular tachycardia; SVES/VES, supra-/ventricular extrasystole; AV block, atrioventricular node block; VT, ventricular tachycardia). **B** Way of admission (EMS, emergency medical services), **C** emergency severity index (ESI) at presentation, **D** reported symptoms at presentation, and **E** patients’ management and further hospital stay. IMC, intermediate care; ICU, intensive care unit; **F** in-hospital therapy according to the initial heart rhythm; and **G** procedures conducted in the electrophysiological examination. (PCI, percutaneous coronary intervention; TAVI, transfemoral aortic valve implantation; eCV, external cardioversion; EE, electrophysiological examination; PM, pacemaker; ICD, implantable cardioverter defibrillator; ER, event recorder; AF, atrial fibrillation; VT, ventricular tachycardia; AV node, atrioventricular node; AVNRT, atrioventricular node re-entry tachycardia; FAT, focal atrial tachycardia; WPW, Wolff-Parkinson-White). Data are presented as percentages

A total of 58% of all patients categorized as CA showed normofrequent SR on the initial ECG. Atrial fibrillation and atrial flutter were most often diagnosed in patients with pathological ECG (Fig. 2A). Baseline characteristics of the patients are shown in Table 1. Patients with pathological ECG were significantly older and suffered more often from cardiovascular comorbidities than patients with normofrequent SR.

**Table 1:** Baseline characteristics

	Patients with SR	Patients with pathological heart rhythm	
***n*** (total)	322	236	
**Age**, mean ± SD	54.4± 20.6	68.9 ± 14.5	***p=< .001***
**Sex male**, %	53.42	57.2	*p=.39*
**Risk factors**	Yes, %		
Diabetes	11.6	19.6	***p=.01***
Hypertension	48.9	69.5	***p=< .001***
Ex-/smoker	25.3	23.6	*p=.68*
Familial disposition	13.2	13.8	*p=.90*
Hypercholesterolaemia	22.8	36.8	***p=.001***
**Antiplatelet therapy at ED admission**	Yes, %		
ASA	23.2	25.5	*p=.54*
ADP-receptor antagonist	6.7	2.6	***p=.04***
DAPT	4.4	1.7	*p=.09*
**Oral anticoagulation/heparin at ED admission** yes %			
VKA	4.8	16.7	***p< .001***
NOAC	8.2	30.9	***p< .001***
Heparin	0.3	0.9	*p=.58*
**Further medication at ED admission**	yes %		
Statin	22.6	31.6	***p=.02***
ACE-inhibitor	17.5	32.6	***p< .001***
Sartan	18.6	22.2	*p=.33*
Calcium antagonist	15.7	16.2	*p=.91*
Diuretic	21.3	42.2	***p< .001***
Beta-blocker	33.7	62.3	***p< .001***
Amiodarone	2.2	2.6	*p=.78*
Flecainide	0.3	3.0	***p=.01***
Other antiarrhythmics	0.3	2.6	***p=.05***
**Coexisting conditions at ED admission**	Yes, %		
History of stroke	6.2	12.0	***P=.02***
History of TIA	0	0.4	*p=.42*
History of CAD	21.8	28.5	*p=.07*
History of CA	29.3	62.7	***p< .001***
History of DCM	3.4	4.2	*p=.66*
**Ejection fraction**	Yes, %		
Normal EF ≥55%	75.0	66.3	*p=.09*
Mildly reduced EF 45–54%	8.9	14.8	*p=.13*
Moderately reduced EF 30–44%	10.7	12.4	*p=.73*
Severely reduced EF < 30%	5.4	6.5	*p=.82*
**Vital signs at ED admission**	Yes, %/mean ± SD		
Temperature	36.8 ± 0.5	36.8 ± 0.6	*p=.30*
Blood pressure			
Hypotensive < 100/60 mmHg	2.5 224 309	5.3 224 309	*p=.11*
Normotensive up to 139/89 mmHg	36.5	46.7	***p=.02***
Hypertensive ≥ 140/90 mmHg	61.0	48.0	***p=.003***
Heart rate			
Bradycardia < 50 bpm	2.3	4.5	*p=.21*
Normal HR 50-99 bpm	80.8	29.9	***p< .001***
Tachycardia > 99 bpm	16.9	65.6	***p< .001***
**Laboratory parameters at ED admission**	Yes (%)/mean ± SD		
Troponin > 0.03 ng/ml	42 (14.58)	47 (22.60)	***p=.02***
CK > 169 U/l	55 (19.23)	50 (23.70)	*p=.27*
CK-MB > 23 U/l	18 (6.38)	21 (10.10)	*p=.18*
ProBNP pg/ml	7960.3±9723.6	2792.0±2549.2	*p=.39*

Clinical baseline characteristics of recruited patients. Vital signs at ED admission: initial measurements taken at presentation to the ED. Data are presented as mean ± SD or percentages, significance levels are indicated. *SR* Sinus rhythm, *SD* Standard deviation, *ED* Emergency department, *ASA* Acetylsalicylic acid, *ADP* Adenosine diphosphate, *DAPT* Dual antiplatelet therapy, *VKA* Vitamin K antagonist, *NOAC* New oral anticoagulants, *ACE* Angiotensin-converting enzyme, *TIA* Transient ischaemic attack, *CAD* Coronary artery disease, *CA* Cardiac arrhythmia, *DCM* Dilated cardiomyopathy, *EF* Ejection fraction, *bpm* Beats per minute

In both groups, the same number of patients was taken to the ED in the ambulance (38.6% in the pathological ECG group vs. 38.2% in SR; *p=0.93*), but more patients with CA (15.3%) than with SR (2.8%) had consulted an outpatient physician before attending the ED (*p<0. 001*; Fig. 2B). Immediately after arrival in the ED, patients with SR were more often triaged with higher ESI scores, indicating a lower urgency of medical treatment (6.4% ESI 4 and 5 in the pathological ECG group vs. 19.6% in SR; *p<0.001*; Fig. 2C). When asked for the main symptom, patients in both groups reported most frequently palpitations and tachycardia making them highly suspicious of suffering from heart rhythm-related disorder leading to the triage code CA. Furthermore, dyspnea and physical weakness were frequently stated by patients with pathological ECG while people with SR more often suffered from chest pain, pre-/syncope/collapse, and gastrointestinal symptoms (Fig. 2D). The initial medical treatment of patients with pathological ECG comprised frequently substitution of electrolytes, e.g., magnesium or potassium and administration of rate-controlling agents like beta-blockers.

### Admitted patients

The admission rate was higher in patients with pathological ECG (60.6%) compared to patients with SR (39.4%) or the total rate of all ED patients (48.0%). However, the rate of patients treated in the intermediate or intensive care unit (Fig. 2E), as well as the total duration of the hospital stay did not differ among groups. None of the patients triaged to CA died.

Eighty-two patients with pathological ECG who were admitted to our hospital underwent invasive investigations. 45.1% of these patients underwent coronary angiography with 14.6% needing a percutaneous coronary intervention (PCI). Thirty-seven of these 82 patients (45.1%) underwent electrical cardioversion, and in 26 patients (31.7%), an electrophysiological examination (EE) was conducted (Fig. 2F). In this cohort, ablation mostly was conducted due to atrial flutter (53.9%) or atrial fibrillation (11.5%, Fig. 2G). One sixth of the patients with SR (*n*=54, 16.8%) underwent invasive investigations. Most of these patients underwent a diagnostic coronary angiography (89%), and in about half of these cases, a PCI was necessary (42.6%). In addition, 16.7% of this group underwent an electrophysiological examination. Here, particularly AV-nodal re-entrant tachycardia/focal atrial tachycardia/Wolff-Parkinson-White syndrome (55.6%) and atrial flutter (33.3%) were treated.

### Discharge diagnosis

The most frequent diagnosis at discharge according to ICD10-codes of the in-hospital patients with pathological ECG was cardiac arrhythmia (69.6%) followed by heart failure (4.8%) and diagnosis subsumed under the topic coronary artery disease (4.0%; Table 2). The majority of patients with SR suffered from cardiac problems as well. Consistent with the initially reported symptoms, they were mostly diagnosed with cardiac arrhythmia although to a significantly reduced extent (29.2%) than patients with pathological ECG. Furthermore, coronary artery disease (19.2%), heart failure (6.7%) as well as infectious (5.9%), and endocrinologic diseases (2.5%) were named in this group.

**Table 2:** Principal diagnosis of in-hospital patients according to the International Statistical Classification of Diseases and Related Health Problems-Codes

	Patients with SR	Patients with pathological heart rhythm	
***n*** (total)	120	125	
**Cardiac diseases,** *n*=yes (%)			
Cardiac arrhythmia	35 (29.2)	87 (69.6)	***p< .001*** **<0,0001**
Coronary artery disease	23 (19.2)	5 (4.0)	***p=.002***
Heart failure	8 (6.7)	6 (4.8)	*p=.59*
Diseases of heart valves and pericardium	6 (5.0)	1 (0.8)	*p=.06*
Hypertension	5 (4.2)	1 (0.8)	*p=.11*
Chest pain	5 (4.2)	0 (0)	***p=.03***
Complication of an implantable device	2 (1.7)	3 (2.4)	*p>.99*
Cardiogenic shock	2 (1.7)	0 (0)	*p=.24*
**Infectious diseases**			
Pneumonia	5 (4.2)	2 (1.6)	*p=.27*
Sepsis	2 (1.7)	2 (1.6)	*p>.99*
**Endocrinologic disease**	3 (2.5)	1 (0.8)	*p=.36*
**Gastroenterological disease**	2 (1.7)	3 (2.4)	*p>.99*
**Haemato-oncologic diseases**			
Tumor	1 (0.8)	1 (0.8)	*p>.99*
Anemia	0 (0)	2 (1.6)	*p=.50*
**Vertigo/syncope**	5 (4.2)	1 (0.8)	*p=.11*
**Lung embolism**	2 (1.7)	0 (0)	*p=.24*
**Others**	12 (10.0)	10 (8.0)	*p=.66*
**No information**	2 (1.7)	0 (0)	*p=.240*

ICD diagnosis of in-hospital patients is shown and correlated to the initial electrocardiogram at admission. Data are shown as numbers and percentages; significance levels are indicated. *SR* sinus rhythm, *ICD code* International Statistical Classification of Diseases and Related Health Problems codes

## Discussion

In this study, we characterized all patients triaged to the code CA in the ED at the Ludwig-Maximilians-University in Munich within 1 year. According to the rhythm detected in the ECG at admission, patients were divided into two groups: normofrequent SR or pathological heart rhythm. More than half of the patients triaged to CA showed SR. Interestingly, the ICD codes at discharge showed that 71.9% of patients in the SR group who were admitted to our hospital suffered from cardiac problems and that 16.7% underwent an electrophysiological examination during their hospital stay. Therefore, the incidence of a pathological heart rhythm might be underestimated in our collective. As a consequence, patients suspected of CA but with normofrequent SR in the ED should be recommended to do an outpatient cardiological follow-up with prolonged ECG monitoring [6]. The fact that a significant number of patients in the SR group already had a history of stroke in the past, emphasizes the importance of a profound work-up to detect paroxysmal atrial fibrillation.

In almost half of all patients, CA of any kind was known before admission to the ED. Especially in the pathological ECG group, the percentage was high at 61.2% indicating the need for better outpatient support for these patients to reduce the need for ED visits and hospital admission rate [7]; even though this might be challenging and time-consuming in some cases. Perhaps new available user-owned devices such as smartwatches can simplify this process [8]. Patients in the pathological ECG group suffered significantly more often from hypertension, diabetes, and hypercholesterolemia than patients in the normofrequent SR group. The Framingham Heart Study identified diabetes, hypertension, and coronary artery disease as risk factors for the development of atrial fibrillation [9]. In our cohort, a trend is shown that patients with pathological ECG also had more often known CAD and were significantly more often affected by hypercholesterolemia, a known risk factor for CAD. This emphasizes the importance of good management of cardiovascular risk factors in primary care to prevent the development of AF in all ages and subsequently reduce ED visits because of AF [10]. However, only intensive outpatient care can reduce the frequency of emergency visits and admission to the hospital for patients with CA. In this manner, resources could be saved, and costs could be reduced [11].

At admission, all patients were categorized into triage codes by an experienced emergency nurse according to the symptoms they described. Despite the expertise of the nurses, this triage system approaches its natural limitations as the symptoms associated with cardiac arrhythmia may vary from individual to individual and overlap with other (cardiac) diseases. In line with this, the ORBIT-AF trial, a multicenter registry of 10,135 outpatients with AF, reported a wide variation in symptoms. Male AF patients reported a lower frequency of palpitations, dyspnea, and chest discomfort compared to female patients. Moreover, 42.5% of men and 31.2% of women were completely asymptomatic [12]. Using this triage system, patients who report symptoms different from palpitations, brady-, or tachycardia might mostly be categorized to another triage code. On the other side, patients with normofrequent SR reported classical symptoms of cardiac arrhythmia, e.g., palpitations to a similar extent as patients with pathological heart rhythm in our analysis. Therefore, the most important finding of this study is that using the patient’s main symptom as a triage code might be more helpful than catch-all terms like CA to improve processes and quality of medical management in the ED. In an upcoming study, we plan to analyze if a triage system focusing on the patients’ symptoms might influence the length of stay in the ED and the rate of admission to the hospital.

As a retrospective analysis, this study has some limitations. Owing to the design of this study, all patients triaged to any other code except CA were excluded from the analysis. Moreover, due to administrative reasons, data of patients who were transferred to other hospitals could not be collected and analyzed. ICD codes of outpatients were also not included in this study. As they are monetarily irrelevant in Germany, they were not audited by health insurance and were thus considered not reliable enough for analysis in our study. The fact that usually rather healthier and younger patients are discharged from the emergency room or transferred to smaller hospitals might therefore shift the proportion of patients with relevant health impairment in this analysis. To analyze the diagnosis of admitted patients at discharge ICD codes were used. Although they are widely accepted, show general validity, and are simply transferable, catch-all terms like “cardiac arrhythmia” associated with ICD codes imply imprecisions.

## Conclusion

Over half of all patients pooled under the code CA in the ED showed SR on ECG at admission. The most abundant arrhythmia was AF. With 49.5%, the hospital admission rate was quite high, but the patients presented to the ED in our cohort were rarely in critical condition. Seventy-two percent of patients admitted to our hospital and presenting with SR had a cardiological diagnosis at discharge. As a high percentage of our cohort had a history of cardiac arrhythmia better outpatient management is needed for these patients to reduce ED visits and save resources.

## Data Availability

The datasets used and analyzed during the current study are available from the corresponding author upon reasonable request.

## Abbreviations

ACE: Angiotensin-converting enzyme
ADP: Adenosine diphosphate
AF: Atrial fibrillation
ASA: Acetylsalicylic acid
AV node: Atrioventricular node
AVNRT: Atrioventricular node re-entry tachycardia
bpm: Beats per minute
CA: Cardiac arrhythmia
CAD: Coronary artery disease
CK: Creatine kinase
DAPT: Dual antiplatelet therapy
DCM: Dilated cardiomyopathy
ECG: Electrocardiogram
eCV: External cardioversion
ED: Emergency department
EE: Electrophysiological examination
EF: Ejection fraction
EMS: Emergency medical services
ER: Event recorder
ESI: Emergency severity index
FAT: Focal atrial tachycardia
ICD: Implantable cardioverter defibrillator
ICD-10: International Statistical Classification of Diseases and Related Health Problems
ICU: Intensive care unit
IMC: Intermediate care
NOAC: New oral anticoagulants
PCI: Percutane coronary intervention
PM: Pacemaker
proBNP: Pro B-type natriuretic peptide
SD: Standard deviation
SR: Sinus rhythm
SVES/VES: Supra-/ventricular extrasystole
SVT: Supraventricular tachycardia
TAVI: Transfemoral aortic valve implantation
TIA: Transient ischaemic attack
VKA: Vitamin K antagonist
VT: Ventricular tachycardia
WPW: Wolff-Parkinson-White

## References

[1] Paw RC (2008). Emergency department staffing in England and Wales, April 2007. Emerg Med J..

[2] Wuerz RC, Milne LW, Eitel DR, Travers D, Gilboy N (2000). Reliability and validity of a new five-level triage instrument. Acad Emerg Med..

[3] https://www.cdc.gov/nchs/data/nhamcs/web_tables/2017_ed_web_tables-508.pdf.: RPKAf. National Hospital Ambulatory Medical Care Survey: 2017 emergency department summary tables. National Center for Health Statistics, 2017. 2017.

[4] Khurshid S, Choi SH, Weng LC, Wang EY, Trinquart L, Benjamin EJ (2018). Frequency of cardiac rhythm abnormalities in a half million adults. Circ Arrhythm Electrophysiol..

[5] Klawki RHMSK (2017). Deutscher Herzbericht.

[6] Schreiber D, Sattar A, Drigalla D, Higgins S (2014). Ambulatory cardiac monitoring for discharged emergency department patients with possible cardiac arrhythmias. West J Emerg Med..

[7] Ptaszek LM, White B, Lubitz SA, Carnicelli AP, Heist EK, Ellinor PT (2016). Effect of a multidisciplinary approach for the management of patients with atrial fibrillation in the emergency department on hospital admission rate and length of stay. Am J Cardiol..

[8] Perez MV, Mahaffey KW, Hedlin H, Rumsfeld JS, Garcia A, Ferris T (2019). Large-scale assessment of a smartwatch to identify atrial fibrillation. N Engl J Med..

[9] Benjamin EJ, Levy D, Vaziri SM, D'Agostino RB, Belanger AJ, Wolf PA (1994). Independent risk factors for atrial fibrillation in a population-based cohort. The Framingham Heart Study. JAMA.

[10] Shah J, Kumar A, Majmundar M, Adalja D, Doshi A, Desai R (2020). Prevalence of cardiovascular risk factors and financial burden in younger adults hospitalized with atrial fibrillation. Heart Lung..

[11] Tang DH, Gilligan AM, Romero K (2014). Economic burden and disparities in healthcare resource use among adult patients with cardiac arrhythmia. Appl Health Econ Health Policy..

[12] Piccini JP, Simon DN, Steinberg BA, Thomas L, Allen LA, Fonarow GC (2016). Differences in clinical and functional outcomes of atrial fibrillation in women and men: two-year results from the ORBIT-AF registry. JAMA Cardiol..

